# ChIPBase v3.0: the encyclopedia of transcriptional regulations of non-coding RNAs and protein-coding genes

**DOI:** 10.1093/nar/gkac1067

**Published:** 2022-11-18

**Authors:** Junhong Huang, Wujian Zheng, Ping Zhang, Qiao Lin, Zhirong Chen, Jiajia Xuan, Chang Liu, Di Wu, Qiaojuan Huang, Lingling Zheng, Shurong Liu, Keren Zhou, Lianghu Qu, Bin Li, Jianhua Yang

**Affiliations:** Key Laboratory of Gene Engineering of the Ministry of Education, State Key Laboratory for Biocontrol, School of Life Sciences, Sun Yat-sen University, Guangzhou 510275, P.R. China; Key Laboratory of Gene Engineering of the Ministry of Education, State Key Laboratory for Biocontrol, School of Life Sciences, Sun Yat-sen University, Guangzhou 510275, P.R. China; Key Laboratory of Gene Engineering of the Ministry of Education, State Key Laboratory for Biocontrol, School of Life Sciences, Sun Yat-sen University, Guangzhou 510275, P.R. China; Key Laboratory of Gene Engineering of the Ministry of Education, State Key Laboratory for Biocontrol, School of Life Sciences, Sun Yat-sen University, Guangzhou 510275, P.R. China; Key Laboratory of Gene Engineering of the Ministry of Education, State Key Laboratory for Biocontrol, School of Life Sciences, Sun Yat-sen University, Guangzhou 510275, P.R. China; Key Laboratory of Gene Engineering of the Ministry of Education, State Key Laboratory for Biocontrol, School of Life Sciences, Sun Yat-sen University, Guangzhou 510275, P.R. China; Key Laboratory of Gene Engineering of the Ministry of Education, State Key Laboratory for Biocontrol, School of Life Sciences, Sun Yat-sen University, Guangzhou 510275, P.R. China; Key Laboratory of Gene Engineering of the Ministry of Education, State Key Laboratory for Biocontrol, School of Life Sciences, Sun Yat-sen University, Guangzhou 510275, P.R. China; Key Laboratory of Gene Engineering of the Ministry of Education, State Key Laboratory for Biocontrol, School of Life Sciences, Sun Yat-sen University, Guangzhou 510275, P.R. China; Key Laboratory of Gene Engineering of the Ministry of Education, State Key Laboratory for Biocontrol, School of Life Sciences, Sun Yat-sen University, Guangzhou 510275, P.R. China; Key Laboratory of Gene Engineering of the Ministry of Education, State Key Laboratory for Biocontrol, School of Life Sciences, Sun Yat-sen University, Guangzhou 510275, P.R. China; Department of Systems Biology, Beckman Research Institute of City of Hope, Monrovia, CA 91016, USA; Key Laboratory of Gene Engineering of the Ministry of Education, State Key Laboratory for Biocontrol, School of Life Sciences, Sun Yat-sen University, Guangzhou 510275, P.R. China; Key Laboratory of Gene Engineering of the Ministry of Education, State Key Laboratory for Biocontrol, School of Life Sciences, Sun Yat-sen University, Guangzhou 510275, P.R. China; Key Laboratory of Gene Engineering of the Ministry of Education, State Key Laboratory for Biocontrol, School of Life Sciences, Sun Yat-sen University, Guangzhou 510275, P.R. China; The Fifth Affiliated Hospital, Sun Yat-sen University, Zhuhai 519000, P.R. China

## Abstract

Non-coding RNAs (ncRNAs) are emerging as key regulators of various biological processes. Although thousands of ncRNAs have been discovered, the transcriptional mechanisms and networks of the majority of ncRNAs have not been fully investigated. In this study, we updated ChIPBase to version 3.0 (https://rnasysu.com/chipbase3/) to provide the most comprehensive transcriptional regulation atlas of ncRNAs and protein-coding genes (PCGs). ChIPBase has identified ∼151 187 000 regulatory relationships between ∼171 600 genes and ∼3000 regulators by analyzing ∼55 000 ChIP-seq datasets, which represent a 30-fold expansion. Moreover, we *de novo* identified ∼29 000 motif matrices of transcription factors. In addition, we constructed a novel ‘Enhancer’ module to predict ∼1 837 200 regulation regions functioning as poised, active or super enhancers under ∼1300 conditions. Importantly, we constructed exhaustive coexpression maps between regulators and their target genes by integrating expression profiles of ∼65 000 normal and ∼15 000 tumor samples. We built a ‘Disease’ module to obtain an atlas of the disease-associated variations in the regulation regions of genes. We also constructed an ‘EpiInter’ module to explore potential interactions between epitranscriptome and epigenome. Finally, we designed ‘Network’ module to provide extensive and gene-centred regulatory networks. ChIPBase will serve as a useful resource to facilitate integrative explorations and expand our understanding of transcriptional regulation.

## INTRODUCTION

Eukaryotic genomes produce various types of RNAs, including mRNAs, microRNAs (miRNAs), long non-coding RNAs (lncRNAs) and other ncRNAs (e.g. tRNAs, snoRNAs). Transcriptional regulation of ncRNAs and protein-coding genes (PCGs) plays an important role in many biological processes, and their dysregulation is frequently involved in the tumorigenesis of many human cancers and the development of various diseases (1,2). Therefore, it is very important to comprehensively decipher the transcriptional regulatory networks between genes and transcriptional regulators, such as transcription factors, histone modifications and chromatin remodeling factors. However, the mechanisms and functions of transcriptional regulation of the majority of genes remain largely elusive.

Chromatin immunoprecipitation followed by sequencing (ChIP-seq) precisely delineates the genome-wide interaction profiles between genes and various transcriptional regulators. Tremendous amounts of ChIP-seq data have been generated by thousands of independent studies and several consortium projects, such as the ENCODE (3) and NIH Roadmap Epigenomics (4) projects, providing new opportunities to explore the transcriptional regulation of various types of ncRNAs and PCGs. A few databases and platforms, such as ReMap (5), CistromeDB (6) and ChIP-Atlas (7), have analysed these ChIP-seq data to explore the binding profiles of transcription factors, histone modification and chromatin remodeling factors. However, these databases are usually limited to the genomic profiles of binding sites, and it is difficult for users to perform rapid association analyses with expression data and various types of gene or disease-related data on a unified platform.

**Figure 1.**
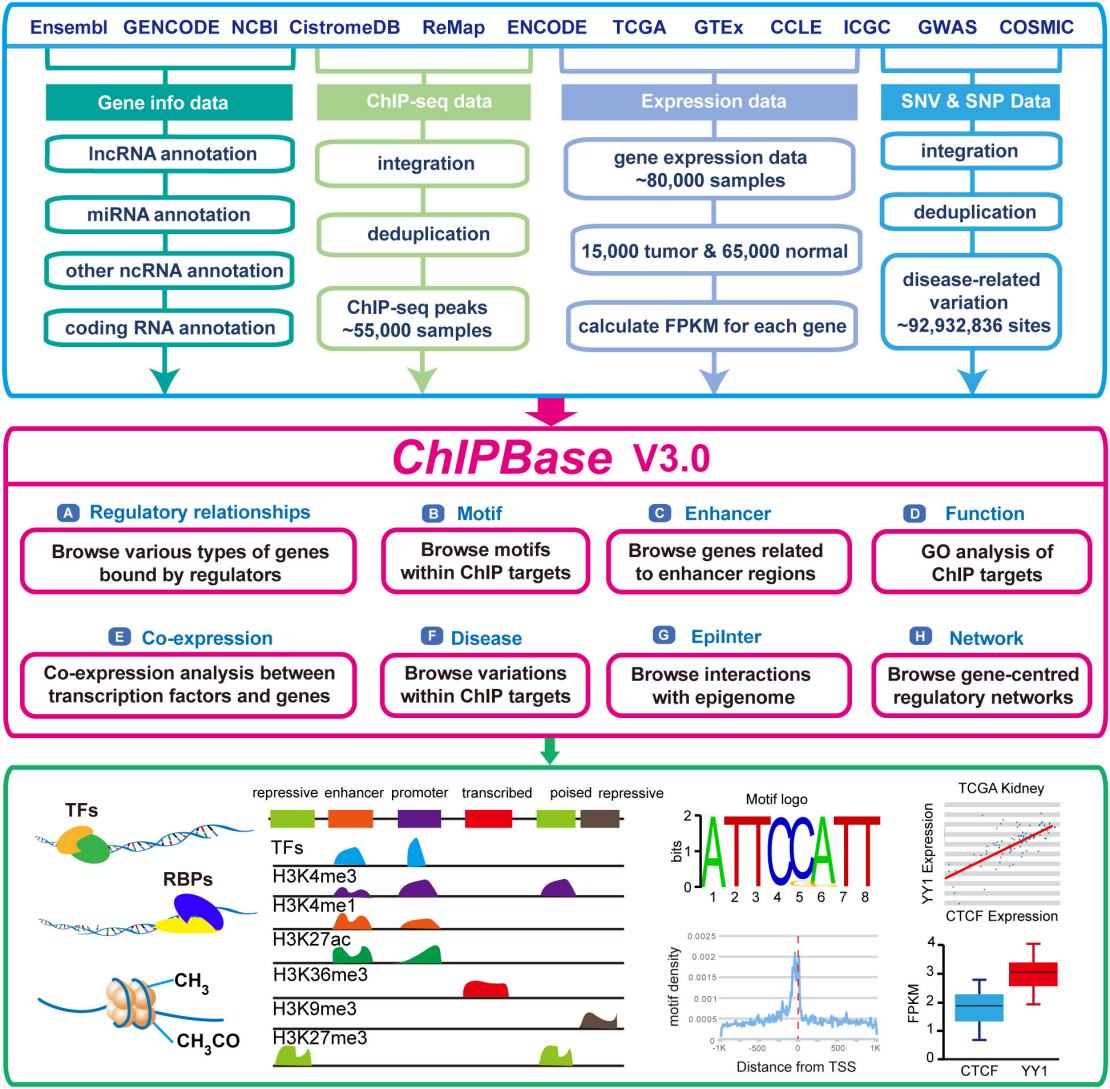
Scheme of the ChIPBase v3.0 framework. A total of ∼55 000 ChIP-seq datasets, ∼80 000 expression datasets, ∼92 932 800 disease-related variation sites and gene data were collected from various resources. All data were further integrated and analysed. All results are stored in MySQL relational databases and displayed mainly in 10 modules on interactive webpages.

To systematically explore the transcriptional regulation of thousands of ncRNAs and PCGs and their relationships with human cancers and diseases, we updated ChIPBase (8) to version 3.0 (Figure 1, Table 1) by integrating huge amounts of ChIP-seq data derived from large consortium projects and public databases. ChIPBase v3.0 constructs millions of regulatory relationships between tens of thousands of ncRNAs or PCGs and thousands of types of regulators by mining ∼55 000 ChIP-seq datasets. ChIPBase v3.0 also provides the comprehensive associations of expression profiles for ncRNAs and their transcriptional regulators in normal and cancer tissues by integrating ∼80 000 RNA-seq data. By integrating millions of SNP and SNV sites, we identified thousands of disease-related regulatory regions of ncRNAs and PCGs. Moreover, ChIPBase v3.0 provides several online analysis tools that focus on exploring the function of regulatory factors, such as the ‘ChIP-co-expression’ and ‘ChIP-GO-function’ tools, by which users can easily perform custom analyses and download the analysis results as well as graphic visualizations. In addition, ChIPBase v3.0 provides a variety of new web modules to perform further analyses and to explore the underlying mechanisms and functions of transcriptional regulators.

**Table 1.** Major improvements of ChIPBase v3.0

Features	ChIPBase v2.0	ChIPBase v3.0
ChIP-seq data	∼10 000	55 420
RNA-seq data	∼20 000	81 701
SNP sites	None	3 993 555
SNV sites	None	88 939 281
genome (human)	GRCh19	GRCh38
genome (mouse)	GRChm10	GRCm39
annotation (human)	Release 25	Release 41
annotation (mouse)	Release M10	Release M30
regulatory relationships (lncRNA)	2 147 760	41 796 020
regulatory relationships (miRNA)	273 760	5 762 250
regulatory relationships (other ncRNA)	4 658 040	12 739 912
regulatory relationships (mRNA)	3 438 820	90 889 229
poised enhancer	None	41 885 621
active enhancer	None	8 975 669
super enhancer	None	141 511
EpiInter module	None	Yes
Enhancer module	None	Yes
Disease module	None	Yes
Network module	None	Yes

## MATERIALS AND METHODS

### Integration of public ChIP-seq data and expression data

We collected a total of ∼55 420 ChIP-seq datasets from the ENCODE project (3), ReMap database (5), CistromeDB database (6) and GEO database (9). We then filtered out low-quality datasets with a peak number of <100. ENCODE provides a variety of processed peak files, but we retained only peaks that had been tested for repeatability. We integrated all the downloaded datasets and removed any redundant records from these datasets. These peak datasets were converted to the corresponding latest genome version by the liftOver tool (10), and the peaks that failed to be converted were discarded. Regarding expression data, 81,701 expression datasets from humans and mice were curated from GTEx (11), ENCODE (3), TCGA (12), ICGC (13) and CCLE (14). Datasets for normal tissue were collected from GTEx and ENCODE, while datasets for cancer were collected from TCGA, ICGC and CCLE. We also filtered out the low-quality datasets with a gene number of less than 200.

### Integration of genome sequences and gene annotation

We downloaded the genome sequences and annotations of five species (human, mouse, worm, fruit fly, *Arabidopsis thaliana*) from GENCODE (15) and Ensembl (16) (Table 1). To analyse the transcriptional regulation of ncRNAs and mRNAs, we extracted and classified genes into lncRNAs, miRNAs, other ncRNAs and mRNAs according to the annotated gene types defined by GENCODE or Ensembl.

### Motif analysis and visualization

We *de novo* identified motifs of transcription factor, histone modification and enhancer regions with the HOMER (17) findMotifsGenome.pl software using the parameters ‘-len 8 -noknown’. We calculated the motif density relative to the transcriptional start site (TSS) with the HOMER findMotifsGenome.pl program using the parameters ‘annotatePeaks.pl tss -size -1000,1000 -hist 10’.

### Transcriptional regulation analysis

We chose 1, 5, 10, 20 and 30 kilobases (kb) upstream and 1, 2, 5 and 10 kb downstream from the TSSs as the examined regulatory domains for various types of genes. Then, we intersected ChIP-seq peak regions with these regulatory domains to generate transcriptional regulatory relationships.

### Coexpression analysis between TFs and various genes

All expression data were normalized by taking log_2_ of the fragments per kilobase million (FPKM) value. Coexpression profiles between ChIP-seq factors and various types of genes were calculated according to the Pearson correlation coefficient (Pearson's *r*) as well as the *P* value in a *t*-test (Student's *t*-test).

### Enhancer identification and classification

It has been reported that poised enhancers are marked with the histone modifications H3K4me1 and H3K27me3, while active enhancers are marked with H3K4me1 and H3K27ac (18). For each aforementioned histone modification, we first computed the *Z*-score of each peak signal to enable comparisons across biological samples. Then, we merged all peak regions within the same condition (e.g. same cell/tissues/treatment) if they overlapped by using the ‘bedtools merge’ program with the parameters ‘-c 4,5,6 -o first,mean,first’. We used the ‘bedtools intersect’ program to find overlap between H3K4me1 and H3K27me3 peaks within the same circumstance for poised enhancer identification. Similarly, we found the overlaps between the H3K4me1 and H3K27ac peaks for active enhancer identification. Based on the active enhancers, we referred to previous methods for identification of super-enhancers (18). If the distance between two active enhancers was less than 12.5 kb, the active enhancers were merged into a longer stitched region. Then, we sorted the stitched and single enhancer regions by their peak scores and generated a curve. The value obtained at the tangent point with a slope value of 1 on the curve was the threshold to distinguish the super-enhancers from the ordinary enhancers. In addition, the length of super-enhancers region had to be >8 kb.

### Identification of disease-related SNPs and SNVs associated with transcription factor binding regions

To investigate the relationships of disease-related SNVs and SNPs with transcriptional regulation, we collected SNP sites from COSMIC (19) and NHGRI GWAS Catalog (20), while SNV sites were collected from COSMIC and ICGC data. As described in our previous study (21), additional SNPs in linkage disequilibrium (LD) with reported disease-related loci were selected with the criteria requiring *r*^2^ > 0.5 in at least one of the four populations (CEU, CHB, JPT and YRI) genotype data of the HapMap project (release 28) (22). All these disease-related SNPs or SNVs were mapped to the extended annotated transcripts and further examined whether they were located in any transcription factor binding regions that contained at least one motif.

### Identification of potential connections between epitranscriptome and epigenome

To investigate the potential connections between epitranscriptome and epigenome, we systematically explored the distributions of RNA modification coordinates around histone modification regions. We downloaded RNA modification sites of humans and mice from the ENCORE databases (23), including m6A, m1A, m5C, m7G, RNA-editing, Nm and pseudouridine sites. We intersected these RNA modification sites with all ChIP-seq peak samples using the ‘bedtools intersect’ program. Finally, we calculated the percentage of interaction in each sample (interaction number/total number).

## DATABASE CONTENT AND WEB INTERFACE

### Exploration of various types of genes bound by transcriptional regulators

To enable extensive exploration of the transcriptional regulation of the genes of interest, ChIPBase v3.0 performs genome-wide analysis of ChIP-seq data with multiple types of genes, including different types of ncRNA genes and PCGs.

Using ChIPBase v3.0, we obtained a total of 171 648 annotated genes from humans, mice, fruit flies, worms, and *Arabidopsis thaliana*. Through conjoint analysis with 55 421 curated ChIP-seq datasets from ∼5000 cell lines or tissue types, we identified ∼41 796 020 transcriptional regulatory relationships between lncRNAs and TFs, ∼5 762 250 between miRNAs and TFs, ∼12 739 900 between other ncRNAs and TFs, and ∼90 889 220 between mRNAs and TFs (Table 1).

**Figure 2.**
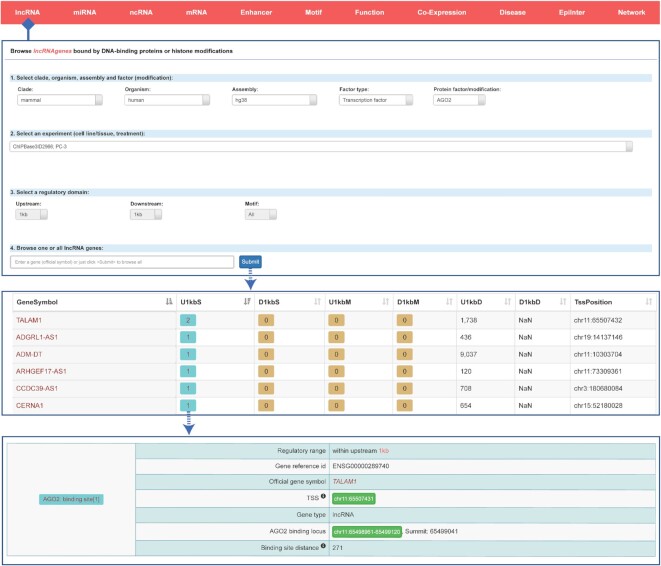
Introduction and use of the ‘LncRNA’ page from ChIPBase v3.0. The submission page for lncRNA genes bound by DNA-binding proteins or histone modifications is shown with its two informational browser pages about selected genes, bound numbers, bound loci and related TSSs.

We provided four web modules for users to browse the transcriptional regulators for lncRNAs, miRNAs, other ncRNAs and mRNAs (Figure 2). Users can select clades, organisms, assemblies and transcriptional regulators of interest, while choose the experiment (cell line/tissue, treatment) and a specific regulatory region. In addition, the user can pull down the ‘Motif’ menu to select motif status, which indicates whether there are any binding motifs of the selected factor within the selected regulatory region. The ‘All’, ‘Y’ and ‘N’ options link to ‘Analyze with or without motifs’, ‘Analyze with motifs’ and ‘Analyze without motifs’, respectively. Finally, users can focus on one or all genes and submit the query information. After submission, the browse pages display genes with their detailed regulatory information, such as the number of binding sites (with/without motif) located within 1 kb upstream or downstream, as well as the nearest distance of binding sites to the TSS of the gene. We also provide an outbound link to a new page showing the detailed information of the binding sites. Users can click the numbers of the binding sites to obtain detailed information about the binding location, related TSS and binding distance.

### Exploration of enhancers located around diverse ncRNAs and PCGs

**Figure 3.**
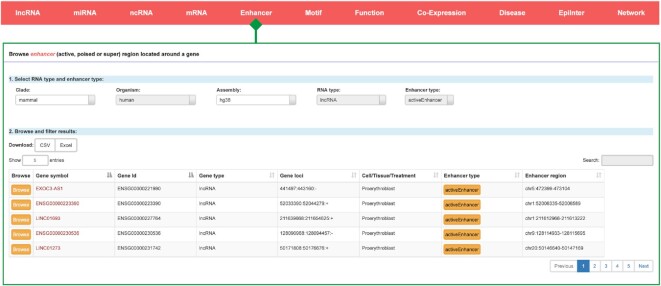
Introduction and use of the ‘Enhancer’ page from ChIPBase v3.0. The browse page for three types of enhancers located around a gene is shown.

Enhancers are crucial cis-regulatory elements for transcriptional regulation and have been extensively studied. To explore more enhancer elements on a large scale, we reanalysed ∼13 232 ChIP-seq datasets of H3K27ac, H3K4me1 and H3K4me3 and identified 41 885 621 poised enhancers, 8 975 669 active enhancers and 141 511 super enhancers from ∼1300 cell lines and tissue types. We further explored the enhancer–gene regulatory pairs within the whole genome and ultimately obtained 54 522 transcriptional regulatory relationships. In the ChIPBase v3.0 release, we constructed a novel ‘Enhancer’ module, in which the user can pull down the ‘enhancer type’ menu to select the poised enhancer, active enhancer or super-enhancer in order to browse the detailed information (Figure 3). The enhancer pages display the enhancer regions related to a specific gene identified from individual conditions. In addition, users can browse the motif information located within the enhancer region.

### Motif profile of DNA-binding proteins around transcriptional start sites

**Figure 4.**
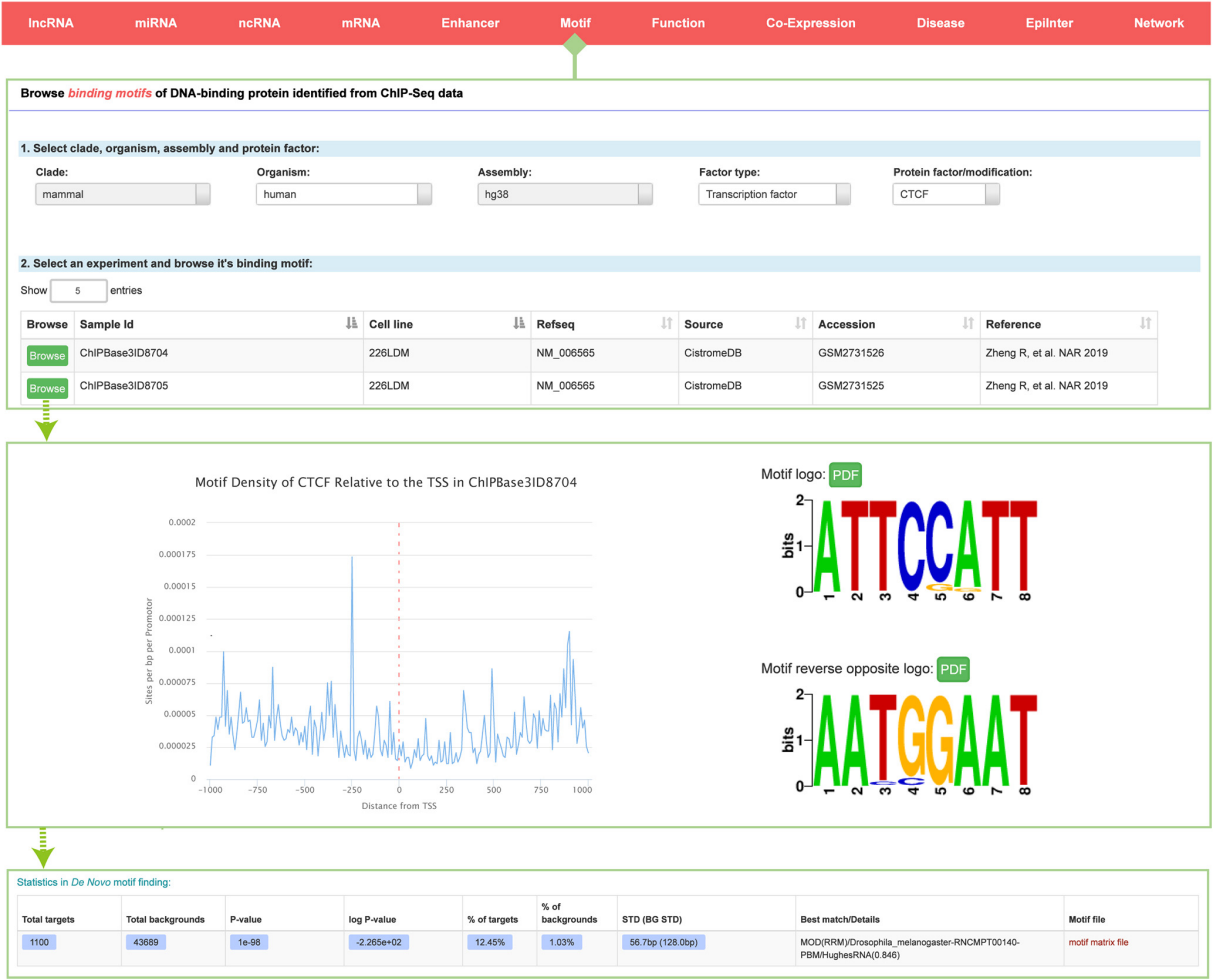
Introduction and use of the ‘Motif’ page from ChIPBase v3.0. The browse page for binding motifs of DNA-binding proteins identified from ChIP-seq data is shown with its two detailed informational pages about motif visualization and statistics.

Motifs are important entry points to study regulatory mechanisms. By integrating large-scale peak data, we *de novo* identified motifs in all transcriptional regulator targets across five species, and ultimately identified 55 247 motifs with significant *P* value. We updated the ‘Motif’ module to provide a more comprehensive overview of the motif information from 28 988 DNA-binding protein datasets in different cell lines and tissues types (Figure 4). All the motif data are displayed as position weight matrices (PWMs), visualized motif logos and binding sites. Users can click the browser button to obtain detailed information, including the p value, percentage and best match to the known motif, and download the motif logo in PDF format for further use. In addition, we offer another section named ‘Motif density relative to the TSS’ to visualize the motif distribution around the gene of interest.

### Web-based tool to analyse biological processes, molecular functions and cellular components for transcriptional regulator targets

**Figure 5.**
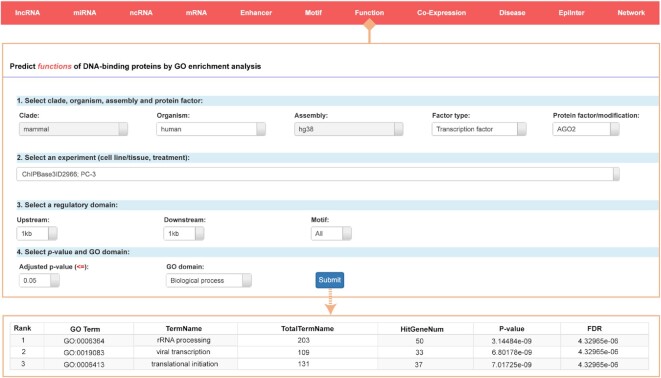
Introduction and use of the ‘Function’ page from ChIPBase v3.0. The submission page for determination of the potential functions of DNA-binding proteins by GO enrichment analysis is shown with an informational browser pages about the GO terms of selected proteins.

The ChIPBase v3.0 platform provides several analysis interfaces for users to explore the functions of different kinds of transcriptional regulators (Figure 5). Users can focus on regulatory regions up to 30 kb upstream and 10 kb downstream of interest, choosing the *P* value threshold and examined GO terms. After the data are submitted, the website will jump to a new result page that displays the detailed GO enrichment results, while a CSV or Excel file containing the results can be downloaded for further study. Users can also search the key words of interest in the result page.

### Exploring the coexpression patterns between transcription factors and genes in diverse normal and tumor tissues

**Figure 6.**
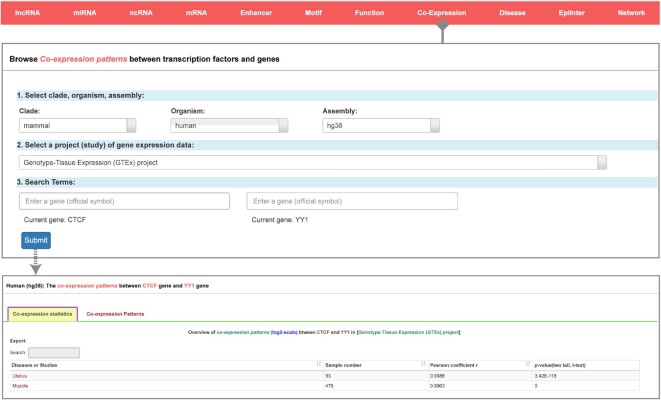
Introduction and use of the ‘Co-Expression’ page from ChIPBase v3.0. The submission page for coexpression patterns between selected transcription factors and genes is shown along with its two detailed informational pages about correlation visualization and statistics.

Coexpression analysis may reveal the potential effects of transcriptional regulators on their target genes. In ChIPBase v3.0, we provide a web-based coexpression tool to explore the coexpression patterns of transcriptional regulators and their target genes via integration of ∼80 000 expression datasets from the ENCODE, TCGA, GTEx, CCLE and ICGC databases, including data for ∼65 000 normal tissue and ∼15 000 cancer samples (Figure 6). Users can select one of the given gene expression datasets, input two genes of interest, and finally click the ‘Submit’ button to explore the coexpression patterns of the queried genes. After submitting data, the user jumps to a new result page that displays the detailed coexpression information, which shows all the types of diseases under study. Users can download a CSV or Excel file containing the results for further exploration. In addition, we offer another section named ‘Co-expression Patterns’ to visualize the correlations between genes. Users can select scatter plots, box plots or histograms to plot the coexpression patterns, which can also be downloaded in various file formats for further use.

### Discovering the transcription factor binding regions associated with disease-related SNPs or SNVs

**Figure 7.**
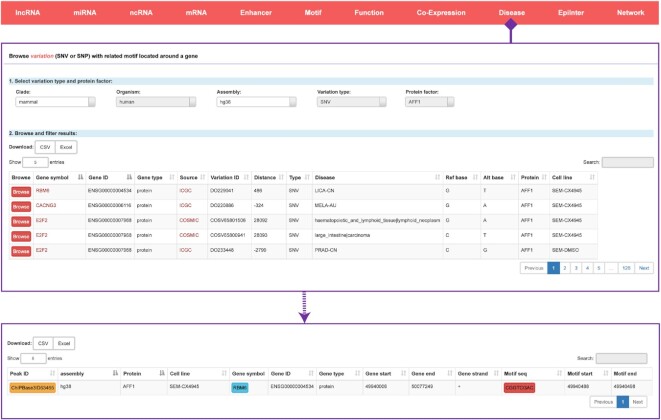
Introduction and use of the ‘Disease’ page from ChIPBase v3.0. The browse page for a variation (SNV or SNP) with the related gene is shown along with a detailed informational page about potential motifs.

To investigate the clinical connections between transcription factors and SNPs as well as SNVs, we systematically explored the distributions of the SNP and SNV coordinates around transcription factor binding regions. We collected 3 993 555 SNP and 88 939 281 SNV sites from the COSMIC, ICGC and GWAS databases and mapped these variation sites to the extended annotated transcripts. We finally identified ∼1 350 000 binding regions with at least one motif around a disease-associated SNP or SNV site. In ChIPBase v3.0, we construct a novel ‘Disease’ module, in which users can select the variation type and transcription factor to browse detailed information of interest, including the variation locus, related disease, altered base, related genes, motif information and so on (Figure 7). We also provided the original SNV/SNP ID and a link to the external database for further exploration.

### Discovering the potential connections between epitranscriptome and epigenome

**Figure 8.**
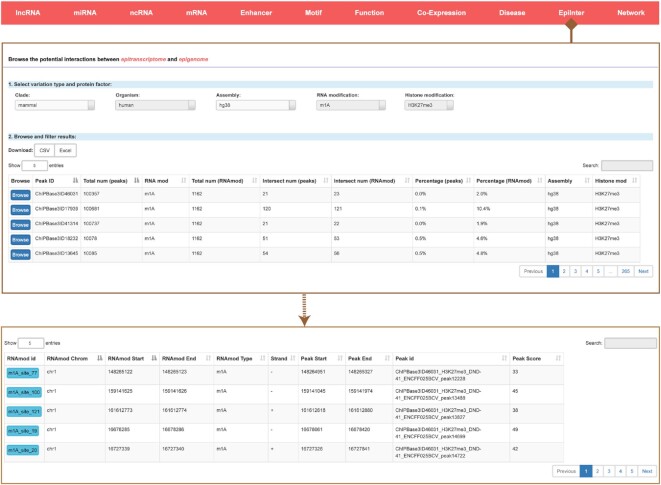
Introduction and use of the ‘EpiInter’ page from ChIPBase v3.0. The browse page for the potential interactions between epitranscriptome and epigenome is shown along with detailed informational pages about all related RNA modification.

Previous studies have shown that significant association occurs between RNA modifications and histone modifications (24–26), such as colocalization of H3K36me3 and m^6^A modifications during transcription (26). To investigate the potential connections between epitranscriptome and epigenome, we systematically explored the distributions of RNA modification coordinates around histone modification regions. We downloaded RNA modification sites of humans and mice from the ENCORE databases and identified 169 263 interaction relationship between ∼150 types of histone modification and 7 types of RNA modification, including m^6^A, m^1^A, m^5^C, m^7^G, RNA-editing, 2’-*O*-methylation and pseudouridine (Ψ). We calculated the percentage of interaction in each sample and provide detailed information about each interaction relationship. In ChIPBase v3.0, we construct a novel ‘EpiInter’ module, in which users can select the RNA modification and histone modification to browse detailed information of interest, including the locus, ChIP-seq coverage and so on (Figure 8). We also provided the original RNA modification ID and a link to the external database for further exploration.

### Exploring the transcriptional regulatory networks of various genes across different samples

**Figure 9.**
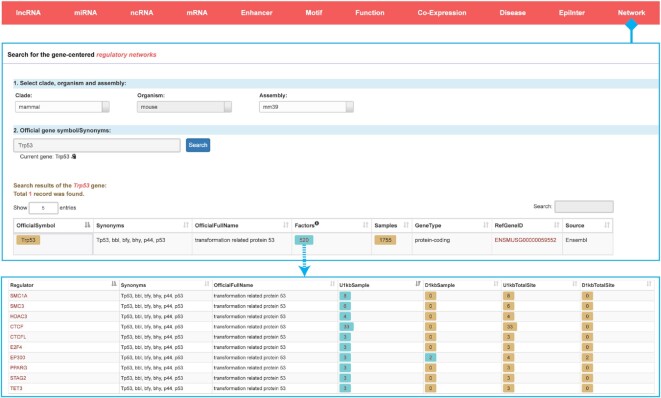
Introduction and use of the ‘Network’ page from ChIPBase v3.0. The browse page for the gene-centred regulatory networks is shown along with detailed informational pages about all related transcriptional regulators.

The expression of a gene is often regulated by many transcriptional regulators. In order to identify all transcriptional regulators of a gene, we constructed the ‘Network’ module to provide extensive and gene-centred regulatory networks (Figure 9). We integrated transcriptional regulators that bound upstream or downstream of various types of genes across different samples. On ‘Network’ webpage, users can search their genes of interest to determine how many transcriptional regulators bind to the genes of interest.

## DISCUSSION AND CONCLUSIONS

In this study, we developed ChIPBase v3.0 to decode the encyclopedia of transcriptional regulation of ncRNAs and PCGs by integrating thousands of multiomics sequencing datasets. Different from other ChIP-seq databases that mainly focus on collecting ChIP-seq data and providing genomic profiles of ChIP-seq peaks, ChIPBase v3.0 has a core theme of transcriptional regulation, as it focuses on research regarding ncRNA and PCG functional networks. Compared with the previous version, ChIPBase v3.0 has significant advances and improvements in many aspects, including data amounts, features and function (Table 1 and Table 2): (a) ChIPBase v3.0 provides the most comprehensive transcriptional regulation atlas of ncRNAs and PCGs by integrating and analysing the peaks downloaded from the GEO database (9), ENCODE project (3), ReMap database (5) and CistromeDB database (6). The amount of ChIP-seq data are increased by up to 5.5-fold in ChIPBase v3.0. This large expansion ensures that analyses of transcriptional regulation can be performed in a variety of cell lines, tissues, and conditions. (b) ChIPBase v3.0 represents a 30-fold expansion of the regulatory relationships between transcriptional regulators and various kinds of genes. This database will provide numerous novel potential functional regions and interaction pairs for the study of regulatory networks. (c) Gene expression data for ∼60 000 genes and clinical matrices of patients from ∼80 000 clinical samples of 32 cancer types and 446 normal or cancer cell lines have been newly added to ChIPBase v3.0. These data have been used to construct coexpression networks; help further reduce the false-positive rates; prioritize the search results in various modules; and facilitate research on the potential functions of transcriptional regulation in developmental, physiological and pathological processes. (d) ChIPBase v3.0 provides ∼29 000 *de novo*-identified binding motifs and high-precision binding maps of DNA-binding proteins. We scanned genome-wide regions upstream and downstream of ncRNAs and PCGs and accurately mapped the distribution profiles to the transcription start sites of genes. In this way, ChIPBase provides researchers with a straightforward way to understand the precise regulatory relationships between TFs and their target genes. In addition, these data will facilitate experimental and computational biologists to correlating their results. (e) ChIPBase v3.0 constructs a novel ‘Enhancer’ module to identify tens of thousands of associations between potential enhancer RNAs (eRNAs) and three types of enhancers from various cell lines or tissues. By enabling a comprehensive characterization of eRNAs in various tissues, we believe that the ‘Enhancer’ module will be a valuable resource for understanding the functions and mechanisms of eRNAs. (f) ChIPBase v3.0, for the first time, links transcriptional regulation to disease development by integrating a large number of disease-related SNV and SNP sites. ChIPBase v3.0 constructs a novel ‘Disease’ module to display the correlations between transcription factor binding regions or motifs and somatic mutations across human tissues and diseases. The vast amounts of variation data collected from ICGC, GWAS, and COSMIC datasets will help researchers to study the influences and functions of transcription factor binding motifs in various genes involved in human diseases. (g) We constructed a ‘Network’ module to provide extensive and gene-centred regulatory networks, allowing researchers to decode more information on transcriptional regulation more quickly for the gene of interest. (h) We also constructed an ‘EpiInter’ module to explore the potential connections between the epitranscriptome and epigenome. (i) We offer the optimized module ‘Batch-download’ to download all files at once more conveniently. (j) ChIPBase v3.0 provides a variety of interfaces and graphic visualizations to facilitate analysis of the massive numbers of DNA-binding protein binding sites, motifs, expression profiles, and regulatory networks in normal tissues and cancer cells. Overall, ChIPBase v3.0 provides biologists with better analysis resources to investigate the transcriptional regulatory mechanisms of ncRNAs and PCGs across different biological conditions that underlie the functions of transcription factors and histone modifications.

**Table 2.** The genome and annotation sources

Species	Assembly	Annotation	Source
*Homo sapiens*	GRCh38	Release 41	GENCODE
*Mus musculus*	GRCm39	Release M30	GENCODE
*Caenorhabditis elegans*	WBcel235	Release 107	Ensembl
*Drosophila melanogaster*	BDGP6.32	Release 107	Ensembl
*Arabidopsis thaliana*	TAIR10	Release 54	Ensembl Plants

## FUTURE DIRECTIONS

With the progress of ChIP-seq technology, an increasing number of types of DNA-level sequencing technologies have been developed and applied, and we hope to collect the resulting new types of data in the future to make our database more complete. We will also integrate the new high-throughput data generated from CLIP-seq and Ribo-seq technologies as well as RNA modification, miRNA target and other data. We will continue to improve the computer server performance for storing and analysing the new incoming data and will continue to develop more tools to conduct a comprehensive analysis of transcriptional regulation.

## DATA AVAILABILITY

ChIPBase v3.0 is freely available at https://rnasysu.com/chipbase3/. The ChIPBase data files can be downloaded and used in accordance with the GNU Public License and the licences of primary data sources.

## Supplementary Material

gkac1067_Supplemental_File
